# Metabolic Intervention with Dimethyl Malonate Impairs Phagocytic Clearance but Fails to Protect Neurons

**DOI:** 10.64898/2026.05.29.724314

**Published:** 2026-06-02

**Authors:** Rachel McNeel, Francisco M. Nadal-Nicolás, Kirsten Overdahl, Wei Li, Alan Jarmusch, Kiyoharu J. Miyagishima

**Affiliations:** 1 National Eye Institute, NIH, Bethesda, MD, USA.; 2 Metabolomics Core Facility, National Institute of Environmental Health Sciences, NIH, Durahm, NC, USA.; 3 Department of Ophthalmology, Faculty of Medicine, University of Murcia and Biomedical Research Institute of Murcia (IMIB-Pascual Parrilla), Murcia 30120, Spain

**Keywords:** retina, thirteen-lined ground squirrel, hibernation, optic nerve crush, dimethyl malonate (DMM), succinate dehydrogenase

## Abstract

Secondary degeneration following optic nerve crush (ONC) is driven in part by mitochondrial dysfunction and microglial activation. Inspired by hibernation, where reduced succinate oxidation limits reactive oxygen species (ROS) production, we tested whether pharmacological inhibition of this pathway confers neuroprotection. Using *in vivo* ONC models and *in vitro* microglial assays, we evaluated the effects of dimethyl malonate (DMM), an inhibitor of succinate dehydrogenase, and a cell-permeable succinate analog (succinate-NV). Succinate-NV increased pro-inflammatory cytokine expression (IL-1β) and reduced anti-inflammatory IL-10, whereas non-permeable succinate had no effect, indicating that intracellular succinate can drive microglial activation. In hibernating animals, succinate-NV disrupted neuroprotection and reduced retinal ganglion cell (RGC) survival following optic nerve injury. Although DMM partially reduced select inflammatory cytokines, it failed to normalize IL-1β or IL-10 and suppressed microglial phagocytosis while exhibiting cytotoxic effects. In vivo, DMM-treated animals showed reduced IBA1 microglia but increased CD68 activation and accumulation of DAPI cells at 7 days post-injury at the crush site. RGC somas persisted but were Caspase3+ consistent with impaired clearance. Astrocyte reactivity increased at lesion borders, while reduced and fragmented GFAP at the lesion site indicated localized astrocyte loss. Collectively, these findings demonstrate that inhibition of succinate oxidation alone is insufficient for neuroprotection and underscore the need for coordinated metabolic and immune regulation that cannot be achieved through single-pathway pharmacological intervention.

## Introduction:

Guided by insights from hibernation, we previously proposed that secondary degeneration arises from mitochondrial-induced activation of retinal microglia (1–4). While these resident immune cells normally function to clear cellular debris (5), overactivation can result in excessive inflammation that may indiscriminately attack stressed or even healthy neurons, compounding the injury. Our prior work suggested that inhibiting succinate oxidation to reduce reactive oxygen species (ROS) production and microglial activation could be beneficial (4, 6).

Although intraocular injection of dimethyl malonate (DMM) mimics the inhibition of succinate oxidation observed during hibernation (4), our current evidence indicates that it is not neuroprotective. While retinal ganglion cell somas appear preserved following ONC, approximately one-quarter of these cells are cleaved caspase-3-positive, and DMM mediated-inhibition of microglial phagocytosis suggests that their persistence reflects failed clearance rather than survival.

## Results:

### Cell-Permeable Succinate Disrupts Hibernation-Induced Neuroprotection

**Figure 1.**
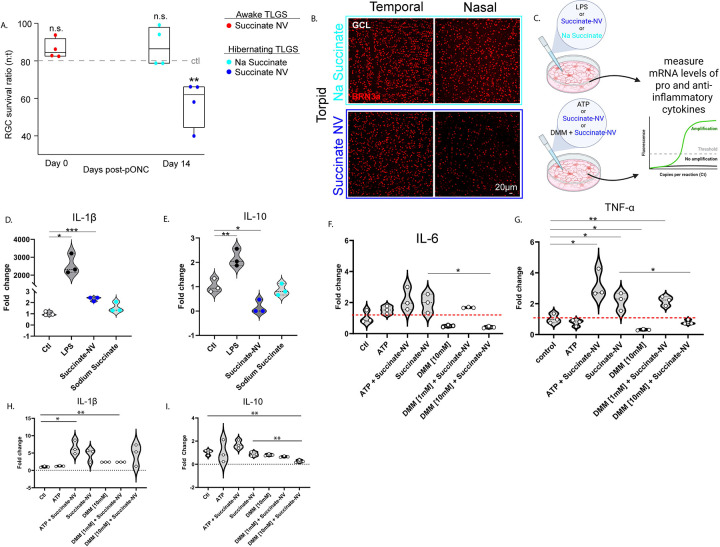
(A) Boxplots showing the RGC survival ratio (nasal:temporal) in awake and hibernating animals following partial optic nerve crush injury. The horizontal black line within each box indicates the median. To investigate the role of succinate in hibernation-mediated neuroprotection, animals received intraocular injections of either sodium succinate (Na Succinate, cell-impermeable) or Succinate-NV (a cell-permeable succinate analog). Succinate-NV injected into naïve awake animals did not induce RGC degeneration. Na Succinate administered to hibernating animals post-ONC did not affect the neuroprotective effect of hibernation. In contrast, Succinate-NV injected into hibernating animals post-ONC significantly reduced RGC survival, indicating that succinate accumulation may contribute to secondary injury mechanisms that compromise cellular viability. (B) Representative high-magnification images of retinal flat mounts from hibernating animals 14 days post-ONC, immunolabeled for BRN3a (red) to visualize RGCs. Images show the nasal (injured) and temporal (uninjured) retina following repeated injections of either Na Succinate or Succinate-NV. (C) Schematic illustrating experimental treatment of BV2 microglial cells in culture. BV2 cells were subjected to lipopolysaccharide (LPS), a cell-permeable succinate analog (Succinate-NV), non-cell-permeable sodium succinate (Na succinate), or dimethyl malonate (DMM) followed by measurement of mRNA levels of inflammatory cytokines. (D,E) Violin plots showing fold changes in mRNA expression of pro-inflammatory cytokine IL-1β (D) and anti-inflammatory cytokine IL-10 (E) in response to 0.1μg/ml LPS, 1.25mM Succinate-NV, or 5mM sodium succinate. 10mM DMM treatment decreases mRNA expression of pro-inflammatory cytokines IL-6 (F) and TNF-α (G) induced by 500μM Succinate-NV. (H) Violin plot showing that Succinate-NV–induced increases in IL-1β mRNA expression at 6 hours were not attenuated by DMM. (I) Violin plot showing fold change in IL-10 expression in BV2 cells treated with Succinate-NV in the presence or absence of DMM. IL-10 expression was suppressed by 10 mM DMM, indicating that DMM exerts context-dependent and not uniformly anti-inflammatory effects. Significant changes indicated by asterisks (* P≤0.05, ** P≤0.01, *** P≤0.001). Error bars indicate standard deviation.

We previously demonstrated that intraocular injection of DMM (0.875 M) inhibits succinate oxidation (4). This mechanism parallels the natural metabolic suppression observed during torpor, where reduced retinal succinate levels limit succinate oxidation and reactive oxygen species (ROS) formation (7). In the current study, we show that intraocular administration of a cell-permeable succinate analog (8) (succinate-NV) in hibernating animals subjected to ONC disrupts RGC survival in hibernating thirteen-lined ground squirrels, as evidenced by a decreased nasal-to-temporal RGC survival ratio (Figure 1A). Importantly, succinate-NV (75 mM) intraocular administration in uninjured, awake animals does not result in RGC degeneration, suggesting that microglia in the absence of injury remain inactive. These findings support the idea that intracellular succinate exacerbates injury-induced microglial activation, thereby contributing to secondary RGC damage. In contrast, intraocular injection of sodium succinate (75 mM)—which is not cell-permeable—has no impact on RGC survival (Figure 1B). This lack of effect is likely due to its inability to enter cells and undergo metabolic oxidation, highlighting the importance of intracellular succinate accumulation in driving microglia-mediated neurodegeneration.

We also performed *in vitro* studies to determine the inflammatory cytokines influenced by Succinate-NV (Figure 1C). Here, lipopolysaccharide (LPS, 0.1μg/ml) was used as a positive control, as it is well known to activate microglia (9, 10) and promote a proinflammatory state (11). The pro-inflammatory cytokine IL-1β was significantly increased in response to both LPS and Succinate-NV, but not to sodium succinate (5 mM) (Figure 1D). In contrast, the anti-inflammatory cytokine IL-10, was decreased by Succinate-NV while remaining unaffected by sodium succinate (Figure 1E).

### Context-Dependent Modulation of Succinate-Driven Inflammation by DMM in Activated Microglia

We then tested whether DMM could counteract the inflammatory changes induced by Succinate-NV. 100 μM ATP was used as a positive control, as ATP is released in the optic nerve in response to injury (12–14), and is known to activate microglia and stimulate cytokine release (15, 16). Interestingly, the addition of 500μM Succinate-NV to ATP-treated BV2 microglia further exacerbated the expression of proinflammatory cytokines, supporting the notion that metabolically primed microglia may be more susceptible to secondary insults that drive them toward an enhanced proinflammatory state. Although DMM showed a dose-dependent ability to reduce IL-6 (Figure 1F) and TNF-α (Figure 1G) it did not rescue the expression levels of IL-1β (Figure 1H) or IL-10 (Figure 1I). In BV2 cells treated with extracellular ATP, a damage-associated molecular pattern (DAMP) signal that triggers inflammatory responses, Succinate-NV significantly increased the proinflammatory mediator IL-1β (Figure 1H). However, the addition of dimethyl malonate (DMM), which is expected to inhibit succinate oxidation, did not significantly reduce IL-1β levels. Furthermore, the addition of Succinate-NV to ATP-stimulated BV2 cells did not alter IL-10 levels (Figure 1I). Paradoxically, DMM reduced the anti-inflammatory cytokine IL-10 in a dose dependent manner. Collectively, these findings suggest that the effects of intracellular succinate and its inhibition by DMM are context-dependent, varying with the mode of microglial activation. Consistent with these findings, another malonate ester prodrug, diacetoxymethyl malonate (MAM), which rapidly releases malonate and inhibits succinate dehydrogenase (SDH) to prevent mitochondrial ROS production (17), also reduced several LPS-induced proinflammatory cytokines in BV2 cells (Figure S1, A-D). Similar to DMM (6), treatment with MAM produced a significant increase in RGC somas compared with untreated retinas; however this does not establish whether these cells remained viable (Figure S2).

### DMM Suppresses Phagocytosis and Exhibits Cytotoxicity Despite Unaltered SDH-Linked Metabolism

**Figure 2.**
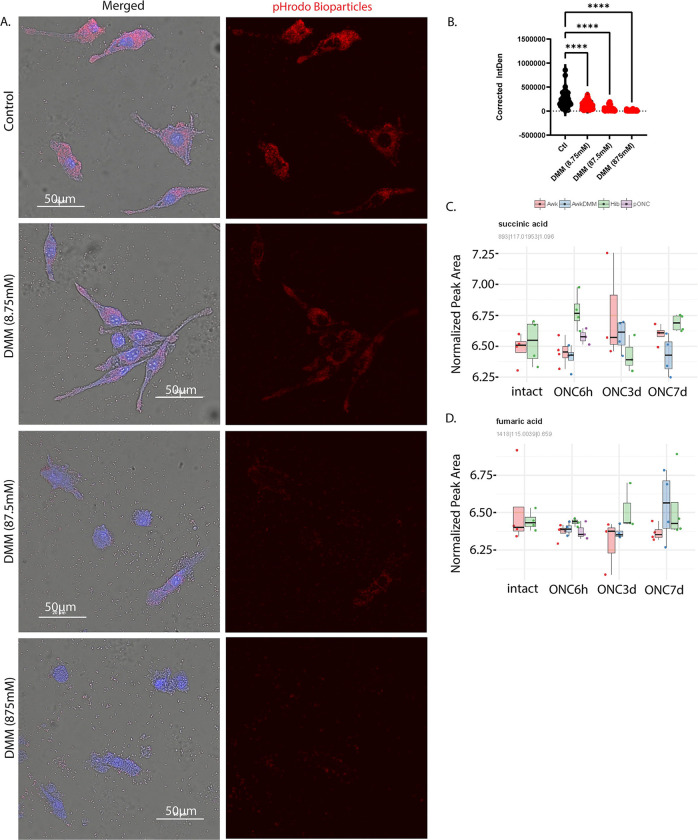
DMM inhibits BV2 microglial phagocytosis in a dose-dependent manner. (A) BV2 microglial cells were pretreated with dimethyl malonate (DMM) for 1 hour prior to incubation with pHrodo^™^ E. coli bioparticles for 2 hours. Internalization of the bioparticles leads to fluorescence within acidic lysosomes, which was reduced in a DMM dose-dependent fashion. These results suggest that DMM impairs microglial phagocytic activity and may hinder clearance of degenerating retinal ganglion cells following optic nerve injury. (B) Quantification of corrected integrated density of internalized pHrodo E. coli bioparticles within microglial cell bodies. Regions of interests (ROIs) were traced along the bright-field images of the BV2 cells and added to the ROI manager in imageJ. The integrated density of the red channel was measured for each cell and background-corrected by subtracting the product of background intensity and cell area. Data represents images from control (n=10), DMM 8.75mM (n=10), DMM 8.75mM (n=9), and DMM 875mM (n=8) groups, corresponding to 48, 66, 55, and 31 cells analyzed, respectively. (C) Negative ion metabolomics analysis of succinate (succinic acid) peak area showing variability between awake and hibernating animals. Following optic nerve injury, DMM treatment did not shift succinate levels in awake animals toward those observed in hibernating animals. (D) Negative ion metabolomics analysis of normalized peak area for fumarate (fumaric acid) also demonstrates variability, with no significant differences observed following DMM treatment compared to awake controls at any post-injury time point. The line in the middle of each box plot represents the median. Box colors indicate squirrel status: Awake (red), Hibernating (green), DMM treated Awake (blue), partial Optic Nerve Crush (pONC) injury (purple). Boxes show the interquartile range and error bars represent standard deviation.

To determine whether the cells were alive, we first considered whether they might simply represent cells that were retained because phagocytosis was inhibited. We found that DMM suppresses microglial phagocytosis in a dose-dependent manner (Figure 2, A–B) and exerts cytotoxic effects in microglial cells, as evidenced by nuclear fragmentation in treated cells. Because DMM inhibits succinate dehydrogenase, the enzyme responsible for oxidizing succinate to fumarate, we next performed metabolomic analyses to assess succinate and fumarate levels in awake and hibernating retinas at multiple time points following optic nerve crush injury. Interestingly, SDH activity, inferred from the relative levels of succinate and fumarate, was not markedly different between awake and hibernating conditions. Following injury, succinate levels exhibited variability but were not significantly different among awake, hibernating, and DMM-treated groups (Figure 2C). Similarly, fumarate levels showed comparable variability and no significant differences across conditions (Figure 2D). Consistent with these findings, the succinate-fumarate ratio-commonly used as a proxy for SDH activity-did not differ substantially between groups, suggesting that ONC does not directly perturb succinate metabolism at a detectable level. However, injury may still alter the broader metabolic environment of the retina and optic nerve in ways that indirectly influence succinate-related pathways, with effects that fall below the detection limits of this analysis.

**Figure 3.**
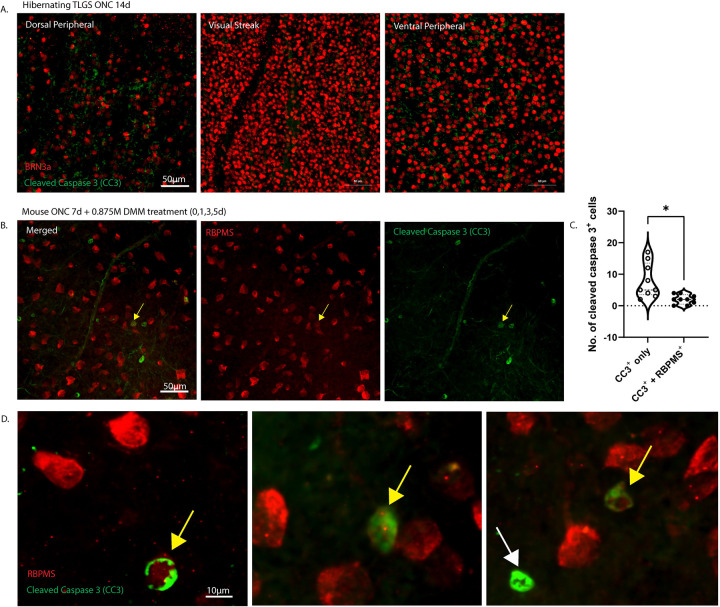
Detection of apoptotic RGC somas in mouse and thirteen-lined ground squirrel retinas using cleaved caspase-3 staining. (A) Hibernating thirteen-lined ground squirrel retinas 14 days after ONC show no co-labeling of RGCs with cleaved caspase-3, suggesting that the cells are not undergoing apoptosis. Note that the thirteen-lined ground squirrel retina exhibits regional variation in RGC density, with the highest densities observed in the visual streak (middle image), followed by the ventral retina (right). (B) Mouse retinas subjected to optic nerve crush (ONC) and treated with dimethyl malonate (DMM; 0.875 M) via intraocular injection on days 0, 1, 3, and 5. Retinal flatmounts collected at day 7 post-ONC were immunolabeled with RBPMS (red) and cleaved caspase-3 (CC3; green). Yellow arrows indicate RGC somas co-labeled with CC3. (C) Quantification of nine images acquired at 20× magnification shows that 25.4% of CC3 cells were RBPMS. There were significantly more CC3 cells alone compared to those co-labeled with RBPMS (p = 0.012). *p < 0.05 indicates statistical significance. (D) Higher-magnification images showing RBPMS RGC somas that are also positive for cleaved caspase-3 (yellow arrows) and cells labeled only with cleaved caspase-3 (white arrow).

Unlike retinas from hibernating thirteen-lined ground squirrel, which showed no evidence of cell death by cleaved caspase-3 staining (18) (Figure 3A) following ONC at 14 days, retinas from DMM-treated mice contained RGC somas that were cleaved caspase-3-positive, indicating ongoing apoptotic signaling despite apparent structural preservation (Figure 3B–D). The incomplete and transient effects of DMM highlight its limited therapeutic potential and reinforce the need to identify more robust metabolic interventions capable of suppressing inflammation and promoting sustained protection to enhance overall cell survival.

### Divergent Microglial and Astrocyte Responses Under DMM Treatment After Injury

**Figure 4.**
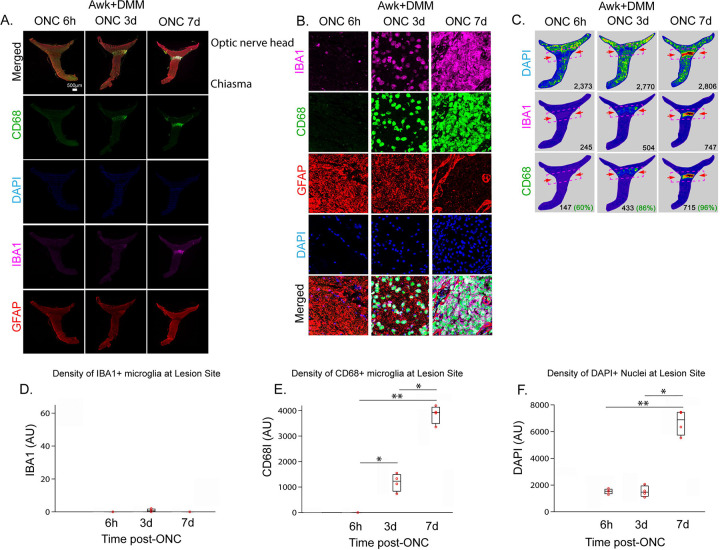
Confocal images of representative optic nerves from DMM-treated thirteen-lined ground squirrel following optic nerve crush, collected at 6 h, 3 d, 7 d post-injury. Optic nerves were stained for CD68 (green), IBA1 (magenta), GFAP (red), and counterstained with DAPI (blue). Scale bar: 500 μm. (B) Representative high-magnification images of the optic nerve crush (ONC) site in Awake DMM-treated animals at multiple time points post-injury (6 hours, 3 days, and 7 days). Optic nerves are immunolabeled for Iba1 (microglia, magenta), CD68 (activated microglia, green), and DAPI (nuclei, blue). Immunostaining reveals dynamic remodeling of the glial environment during the progression of injury and repair. Scale bar: 50 μm. (C) Representative isodensity plots showing the spatial distribution of DAPI, Iba1, and CD68 immunolabeling across the different time points post-injury. Each isodensity plot represents the density of the immunofluorescence marker indicated in the bottom left corner of the first image on the left. The top row shows DAPI (nuclei) density, the middle row shows Iba1 (microglia) density, and the bottom row shows CD68 (activated microglia) density. The region of the crush site, indicated by red arrows, is delineated by a red dashed box superimposed on the isodensity plot. (D) Quantification of IBA1 CD68 microglia at the lesion site reveals no change, limited numbers following ONC. (E) In contrast, CD68 microglia show a progressive increase over time post-injury. DAPI nuclei count subsequently increases by 7d as cells infiltrate the lesion site. Significant changes are denoted by asterisks (*P ≤ 0.05, **P ≤ 0.01). Error bars indicate standard deviation.

Thus, it is unsurprising that DMM does not confer neuroprotection *in vivo*. In squirrels treated with DMM 2 days prior to optic nerve injury, followed by treatment on the day of injury and every other day for up to 7 days, the number of IBA1 microglia remains low and largely unchanged compared to 6 hours and 3 days post-injury (Figure 4A, 4D), likely reflecting DMM-induced toxicity. Despite this, there is a steady increase in CD68 cells at the lesion site by 7 days (Figure 4A, 4C, 4E). This increase is likely driven by the activation of surviving microglia in response to injury, combined with ongoing microglial turnover due to DMM toxicity.

In contrast to the earlier microglial response, astrocyte activation occurs on a slower timescale. By 7 days post-injury, astrocytes outside the injury site become reactive in response to persistent injury signals (e.g. ATP and inflammatory cues) (Figure 4A). Impaired clearance and sustained damage likely further amplify this response. However, at the crush site itself, GFAP staining is reduced and appears discontinuous. This likely reflects local tissue damage caused by the mechanical injury, resulting in loss of viable astrocytes. The punctate, fragmented GFAP signal is consistent with cytoskeletal breakdown as astrocytic processes degenerate or retract.

Consistent with impaired clearance, the number of DAPI cells at the lesion site are elevated at 7 days post-injury (Figure 4F). Together, these findings suggest that although DMM interferes with the clearance of dying RGCs, surviving microglia remain activated and accumulate at the lesion. Concurrently, astrocyte reactivity is more pronounced at lesion borders, where increased GFAP expression reflects a transition from acute immune response to a more structural, glial scar-associated phase. This shift may be further exacerbated by impaired phagocytic clearance following injury.

## Discussion:

Although DMM functions as a metabolic modulator by inhibiting succinate oxidation, it fails to reproduce the broader protective phenotype associated with hibernation following optic nerve injury. While this intervention partially mimics the inhibition of succinate dehydrogenase and suppression of mitochondrial ROS production observed during torpor, our findings demonstrate that this single-pathway approach is insufficient to confer neuroprotection in response to injury-induced ATP release.

A key limitation of DMM appears to be its impact on microglial function. DMM exhibits cytotoxic effects, suppressing microglial phagocytosis and resulting in reduced IBA1 microglial numbers but a paradoxical increase in CD68 cells at later time points. This suggests that although microglial abundance is diminished, surviving cells remain activated. Importantly, the persistence of approximately 25% cleaved caspase-3–positive RGC somas indicates that apoptotic processes continue despite apparent structural preservation, consistent with impaired clearance rather than true neuronal survival.

In vivo, this dysregulation is further reflected by the persistence of retinal ganglion cell bodies, supporting the idea that DMM disrupts efficient removal of RGC somas. This failure of clearance likely sustains local inflammatory signaling, contributing to prolonged activation of glial cells. While early microglial responses are altered by DMM treatment, astrocyte activation emerges at later stages, with increased GFAP expression observed at the lesion borders by 7 days post-injury. In contrast, reduced and fragmented GFAP staining inside the lesion site likely reflects astrocyte loss and cytoskeletal disruption due to mechanical damage and ongoing degeneration.

Interestingly DMM’s effect on microglial cells appears to be context dependent and influenced by the mechanism of microglial activation. LPS binds to toll-like receptor 4 (TLR4)(19, 20), which is known to activate downstream NF-KB signaling pathways (21). This leads to ameboid activation of microglia and enhanced phagocytosis (20, 22). This innate immune response differs from that elicited by the release of damage-associated molecular patterns, such as ATP (23, 24), from injured RGCs and astrocytes (25). Extracellular ATP acts as a “find me” signal to microglia (26), promoting their recruitment and activation through purinergic receptors. This, in turn, stimulates the release of neurotoxic factors such as IL-1β (27) and TNF-α (28), which drive neurodegeneration (12, 29). Based on our results, DMM does not appear to alter the microglial response to injury in the same manner as hibernation does.

Together, these findings highlight a critical distinction between metabolic suppression and functional neuroprotection. Although hypothermia-induced reductions in metabolic demand are known to confer a degree of neuroprotection, the magnitude of protection often greatly exceeds what would be expected from metabolic suppression alone, suggesting that additional mechanisms contribute to the protective phenotype (30, 31). Furthermore, while conventional reasoning predicts that reduced metabolism should simply suppress neuronal activity, experimental studies have demonstrated that direct inhibition of neuronal activity under hypoxic conditions can paradoxically exacerbate irreversible tissue damage (32, 33). These observations indicate that neuroprotection cannot be fully explained by metabolic depression alone.

In the hibernating state, reduced succinate oxidation occurs within a coordinated metabolic framework that preserves cellular viability while maintaining effective immune regulation. In contrast, pharmacological inhibition with DMM disrupts this balance, impairing essential microglial functions such as phagocytosis while failing to adequately suppress inflammatory activation. These results suggest that successful neuroprotective strategies will require a more comprehensive approach that integrates metabolic regulation with preservation of glial function and tissue homeostasis. Future studies aimed at defining the broader metabolic adaptations that occur during hibernation and the injury response—particularly those governing immune cell function and debris clearance—may provide more effective targets for therapeutic intervention following CNS injury.

## Methods:

### Study design

Retinas were collected from naïve mice and from mice following optic nerve crush injury as well as awake (summer active) squirrels subjected to optic nerve injury and treated with DMM pre and post injury for up to 6h, 3d, or 7d.

### Animals

All animal procedures were conducted in accordance with U.S. federal regulations and the ARVO Statement for the Use of Animals in Ophthalmic and Vision Research. Experimental protocols were approved by the National Eye Institute (NEI) Animal Care and Use Committee ASP-606 (mouse) and ASP-595 (squirrel). Adult male and female thirteen-lined ground squirrels (thirteen-lined ground squirrel; *Ictidomys tridecemlineatus*), aged 6–12 months, were obtained from the University of Wisconsin–Oshkosh and housed at the NIH under seasonally adjusted light–dark cycles mimicking natural photoperiods. For squirrels, euthanasia was performed by induction of anesthesia using isoflurane in an induction chamber placed inside a fume hood. Deeply anesthetized animals were euthanized by rapid decapitation using a guillotine. Adult C57bl6 (WT) mice 4–8 months old were also used in this study. Mice were euthanized by CO_2_ inhalation. Animals were placed into an uncharged chamber, and CO_2_ was introduced at a rate of 30–70% of the chamber volume per minute. The CO_2_ concentration gradually increased until animals were anesthetized, after which the flow was increased to achieve euthanasia by overdose. Animals were monitored for cessation of respiration, and death was confirmed by cervical dislocation.

### Optic Nerve Crush (Squirrels)

Squirrels were anesthetized with isoflurane (5% induction, 4% maintenance) delivered via a nose cone, with waste gas scavenged using an activated charcoal filter. Sterile instruments were autoclaved and disinfected between animals. Topical antibiotic ointment was applied to the surgical eye, and lubricating gel was used to prevent corneal dehydration. Ketoprofen (5 mg/kg, s.c.) was administered for analgesia prior to recovery. Normothermic animals were maintained on gentle external heat. Under a stereomicroscope, a temporal canthotomy and lateral conjunctival incision were performed to expose the optic nerve. The retinal blood supply was preserved. The optic nerve, which is horizontal in squirrels (34), was isolated by blunt dissection and crushed using calibrated cross-action forceps for 10 seconds at 1–2 mm posterior to the globe. Following injury, the surgical site was sutured, and animals were monitored until fully ambulatory. Postoperative monitoring included assessment of pain and distress, with veterinary intervention as needed. Animals were euthanized at 6 hours, 3 days, or 7 days post-injury for anatomical assessments.

For partial optic nerve crush, the crush site was positioned along the horizontal optic nerve such that only the nasal portion of the optic nerve was injured. This resulted in selective damage to the nasal retina while sparing the temporal retina, which served as an internal control.

### Optic Nerve Crush (Mice)

Mice were anesthetized with an intraperitoneal injection (IP) of ketamine and xylazine. A drop of proparacaine is provided to the eye as a topical anesthetic. A small incision is made in the dorsal conjunctival membrane temporally. Forceps are used to retract and expose the optic nerve intra-orbitally. While retracting the eye, a curved forceps is used to crush the optic nerve for 10s, approximately 1–2mm behind the optic disc. Care is taken not to damage the blood supplies. Following the procedure, poly-neo-bac is applied to the eye, and the animal is placed back into their cage on a heating pad. The animal is monitored until it is ambulatory. Ketoprofen is provided post-surgery to relieve pain and inflammation.

### Succinate-NV Preparation and Intraocular Injection

Succinate-NV, a cell-permeable prodrug (Oroboros Instruments, Cat#: 60201–01), was prepared as follows. Twenty-five milligrams of Succinate-NV were dissolved in 95.3 μL of DMSO to generate a 1 M solution, which was further diluted 1:1 in DMSO to yield a 500 mM stock. Aliquots (30 μL) were prepared and stored at −80 °C, where they remained stable for up to 4 months per manufacturer’s guidelines. For injections, a single 30 μL aliquot was thawed immediately prior to use and diluted with 170 μL of sterile PBS to obtain a 75 mM suspension. The solution was maintained at ~25–30 °C to prevent precipitation and vortexed immediately before injection.

Hibernating 13-lined ground squirrels (thirteen-lined ground squirrel) received intraocular injections of 15 μL per eye using a 30-gauge Hamilton syringe (Hamilton Co., NV). To facilitate entry, a 27-gauge needle was first used to create a scleral puncture site. Injections were administered one day before partial optic nerve crush (ONC; Day −1), on the day of ONC (Day 0), and again on Days 4 and 9 post-ONC. Retinas were harvested on Day 14.

The intraocular volume of thirteen-lined ground squirrel eyes was experimentally determined by removing the vitreous and measuring its volume in an Eppendorf tube against known volumes. The vitreous volume was estimated to be ~280 μL. Based on this measurement, the final intraocular concentration of Succinate-NV following injection was calculated to be approximately 4 mM.

### Sodium Succinate Preparation (intraocular injection)

Sodium succinate dibasic hexahydrate (25.7 mg; Sigma, Cat#: S9637) was dissolved in 95.3 μL of sterile water (KD Medical, Cat#: RGF-3410) to prepare a 1 M stock solution. Note: sodium succinate is insoluble in DMSO. The solution was further diluted 1:1 with 95.3 μL of sterile water to obtain a 500 mM stock. For working solutions, 15 μL of the 500 mM stock was diluted into 100 μL of sterile PBS to yield a final concentration of 75 mM.

### Immunohistochemistry: Retina

Eyes were fixed in 4% paraformaldehyde (PFA) for 1 hour and dissected in PBS. The anterior segment and lens were removed, and eyecups were cut into four radial petals. Retinas were carefully isolated from the retinal pigment epithelium and optic nerve head, and the vitreous was removed. Retinas were permeabilized and blocked in 2% normal goat serum (NGS) with Triton X-100 in PBS for 30 min. Tissues were then incubated at 4 °C for 7 days with primary antibodies against Brn3a (1:750, Santa Cruz) or RBPMS (1:500, Genetex). Following PBS washes, retinas were incubated with Cy3-conjugated donkey secondary antibodies overnight at room temperature.

### Immunohistochemistry: Optic nerve

Optic nerves were fixed in 4% PFA for 1 hour, washed in PBS, embedded in OCT, and cryosectioned at 30 μm. Sections were incubated with antibodies against CD68 (1:100, Bio-Rad), IBA1 (1:100, Wako), and GFAP (1:500, Aves Labs). Nuclei were counterstained with DAPI (1:2000). Sections were mounted with antifade medium and imaged using a Zeiss LSM 780 confocal microscope.

### Gene expression analysis

RT-qPCR was used to quantify gene expression changes in BV2 microglial cells. BV2 microglial cells were treated with various reagents (e.g., LPS, Succinate-NV, Sodium succinate, ATP, DMM) for 6 hours, after which cells were collected for RNA extraction and subsequent RT-PCR analysis to quantify gene expression changes. Total RNA was extracted using NuceloSpin RNA, Mini kit for RNA purification (Takara Bio, Cat: 740955.50) and reverse-transcribed to cDNA using (Takara PrimeScript 1^st^ strand cDNA synthesis kit (Takara Bio, Cat: 6110A) according to manufacturer’s instructions. cDNA was used for RT-qPCR in a reaction volume of 15μl containing, 2μL cDNA, 2μl of primer mixture, and 7.5 μl of Master mix (EmeraldAmp^®^ GT PCR Master Mix, Takara Bio, Cat: RR310B) and 3.5 μl of water. RT-qPCR was performed using a CFX96 Real-Time PCR Detection System (Bio-Rad). Ribosomal protein S14 (RPS14) was used as an internal control. mRNA expression levels were normalized to control samples using the comparative Ct (2^−ΔΔCt^) method. A list of primers is shown below. At least 3 replicates of each experiment were performed.

Primers:

**Table:** 

TNF-α-662	CCAGGAGAAAGTCAACCTCC
TNF-α-870	GAGCAATGACTCCAAAGTAGAC
IL-10-439	GCGCTGTCATCGATTTCTCCCC
IL-10-640	TGGAGTCCAGCAGACTCAATACACA
IL-1β-662	GCTGGAGAGTGTGGATCCC
IL-1β-807	TGTGCTCTGCTTGTGAGGTGCTG
IL-6-450	GAGTCCTTCAGAGAGATACAG
IL-6-667	CTAGGTTTGCCGAGTAGATC
RPS14-548	CACAGACGGCGACCACGAC
RPS14-363	CACTGCCCTGCACATCAAACT

### Untargeted Metabolomics Sample Extraction

Samples were prepared first by homogenizing via bead-beating (Omni Hard Tissue Homogenizing Mix 2.8 mm ceramic (2 mL tubes), SKU 19–628; 50 mg biological material:1mL water; Omni Ruptor Bead Elite, 2.6 m/s, 30 seconds, 1 cycle); protein precipitation was then performed by adding ice-cold methanol (Fisher Chemical, Optima^™^ LC-MS grade) to homogenate (4:1 v/v). Samples were vortexed and placed in a −20°C freezer for 30 min to facilitate precipitation; samples were then centrifuged and 900uL of supernatant was transferred to a new 1.5 mL microcentrifuge tube. Samples were dried (Genevac EZ-2 Plus, 40°C, 2hr.) and resuspended via the addition of 200 microliters of water-acetonitrile 98%:2% v/v. An aliquot of each samples was transferred to a 2mL autosampler vial with microvolume insert (Agilent); an aliquot from each extract was additionally transferred into a new 1.5 mL microcentrifuge tube to create a pooled quality control sample.

### Untargeted Metabolomics Analytical Measurement

Samples were analyzed using an ultra-high performance liquid chromatograph (UHPLC, Vanquish, Thermo Scientific) coupled to a high-resolution mass spectrometer (Orbitrap Fusion Tribrid, Thermo Scientific) via heated electrospray ionization (NG Ion Max, Thermo Scientific) in positive and negative modes. UHPLC was used to separate chemicals, prior to mass spectrometric analysis, based on physical and chemical characteristics such as polarity. MS and MS/MS data were acquired. LC-MS data were collected from individual samples (n = 1 injection), system blanks (injection of solvent used to resolubilize samples), and a pooled quality control. The pooled quality control (QC) was injected multiple times at different volumes and used in data processing. MS/MS data, used to annotate features, were collected using the AcquireX (Thermo Scientific) deep scan methodology in which pooled QC was injected multiple times (n = 7). Chromatographic separation was carried out on a 2.1 × 100 mm, 100Å, 2.6 μm, F5 analytical column (Phenomenex) with corresponding guard cartridge. Gradient elution was performed using water with 0.1% acetic acid v/v and acetonitrile with 0.1% acetic acid v/v.

### Untargeted Metabolomics Data Processing

Compound Discoverer 3.3.0.550 (Thermo Scientific) was used to process data files which resulted in a tabular output of descriptors of each feature (e.g. m/z, retention time), annotation information (e.g. MS/MS database match), and peak area. We processed the output from Compound Discoverer using in-house R scripts via JupyterNotebooks. The major components of the processing included formatting of the data outputs, comparison of m/z and retention time of annotation features versus an in-house generated list based on authentic chemical standards, assessment of signal response in pooled QC samples, assessment of signal variance in pooled QC samples versus samples (i.e. dispersion ratio), and multi- and univariate statistics. Features were annotated via spectral database matching to in-house libraries, NIST libraries, GNPS libraries, and m/zCloud libraries.

### Statistical analysis

Statistical analyses were performed using GraphPad Prism, Microsoft Excel, or custom MATLAB scripts. Pairwise comparisons were conducted using paired t-tests, while comparisons among three or more groups were performed using one-way analysis of variance (ANOVA).

## Supplementary Material

Supplement 1

## Data Availability

The datasets supporting the conclusions of this article are included within the article (and its Supplementary Material). Additional inquiries may be directed to the corresponding author.
