# Supplementary material for: Metabolic Intervention with Dimethyl Malonate Impairs Phagocytic Clearance but Fails to Protect Neurons
Source: bioRxiv. 2026 Jun 2:2026.05.29.724314. Preprint. [Version 1] doi: 10.64898/2026.05.29.724314 (PMC13251941; doi:10.64898/2026.05.29.724314)
Supplement: Supplement 1 [file media-1.docx]

## Supplementary Information


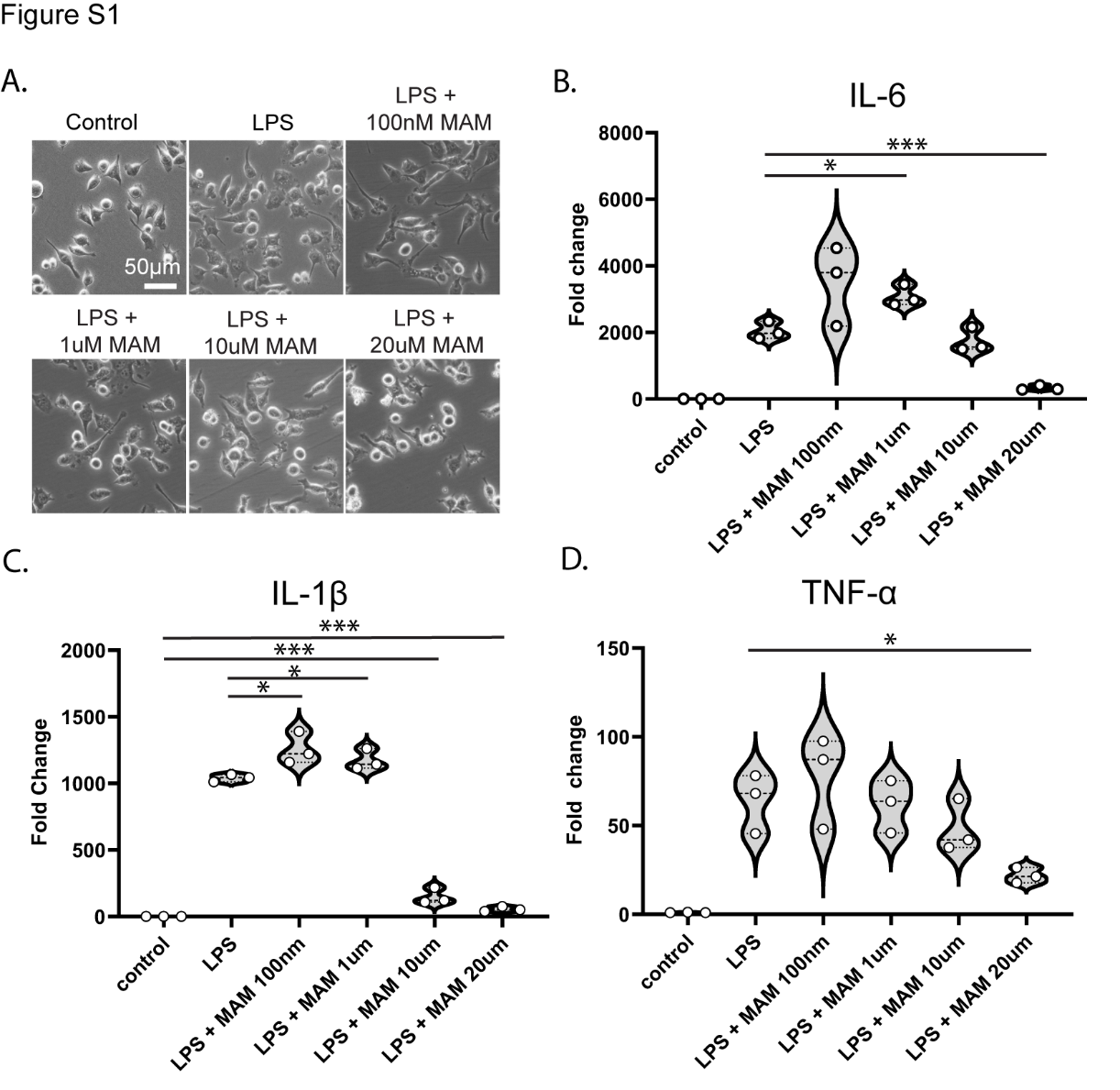


**Supplementary Figure S1.** Changes in mRNA expression of inflammatory chemokines following 6h treatment with LPS ± MAM. (C) Phase-contrast images of BV2 cells after 6 h of LPS stimulation in the presence of increasing concentrations of MAM, a malonate prodrug that—similar to DMM—inhibits succinate dehydrogenase. (D-F) Violin plots showing fold change in inflammatory cytokine expression in response to LPS stimulation ± MAM treatment. (D) LPS stimulation increased IL-6 expression, which was suppressed in a dose-dependent manner by MAM. (E) LPS induced IL-1β expression in BV2 microglial cells, which was attenuated by MAM at concentrations ≥ 10 mM. (F) LPS stimulation increased TNF-α expression, which also showed a dose-dependent decrease with MAM treatment.


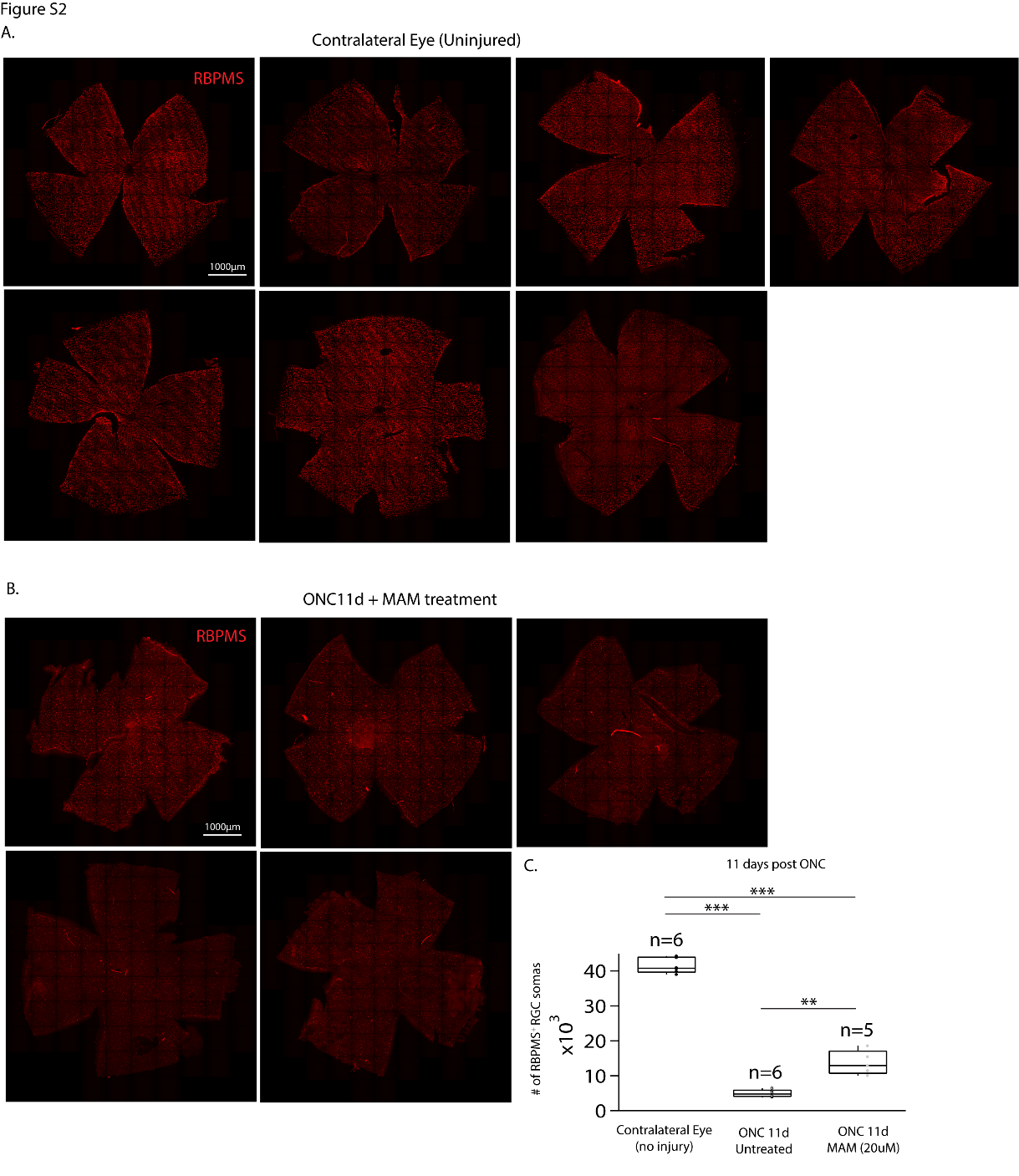


**Figure S2.** Immunofluoresecent images of mouse retinas 11d post optic nerve injury. (A) Retinas from Contralateral (uninjured eye, n=7) is shown for comparison (1) along with (B) optic nerve injury (treated with MAM 20uM, n=5). Contralateral eye (39766.4+/-1141.7) RGCs, ONC11d with MAM-treatment (13678+/-1516.1) (D) Boxplot showing the number of RGC somas remaining at 11d post ONC. Error bars represent standard deviation.

**
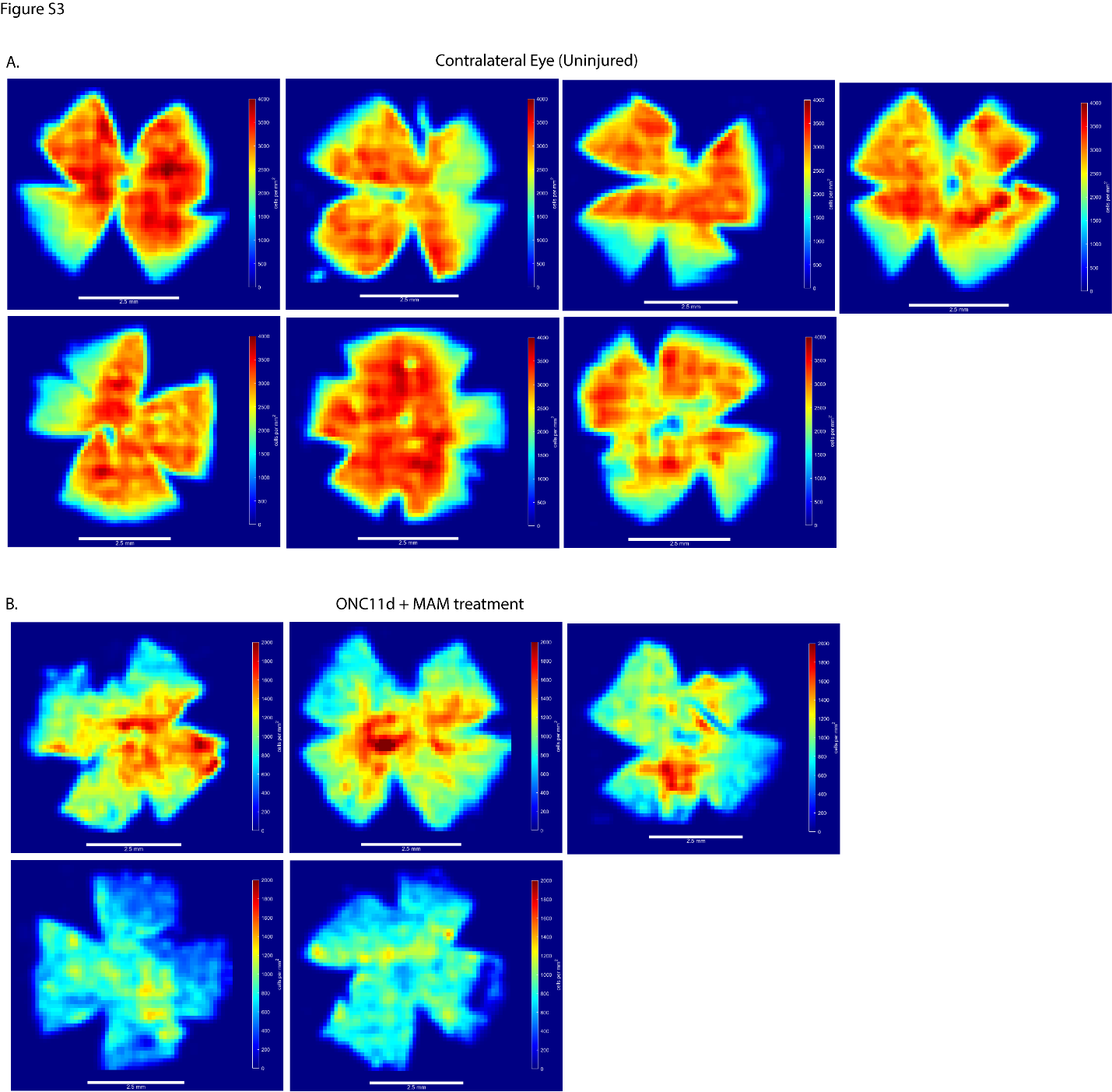
**

**Figure S3.** Isodensity plots of images of mouse retinas 11d post optic nerve injury. (A) Retinas from Contralateral (uninjured eye, n=7) is shown for comparison along with (B) optic nerve injury (treated with MAM 20uM, n=5).
